# The Experience of Depression, Self-Efficacy, and Family Functioning Among Mothers of Children with Autism Spectrum Disorder: A Mixed Methods Study 
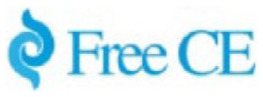


**DOI:** 10.1177/10783903261417989

**Published:** 2026-02-14

**Authors:** Stefanie Zavodny Jackson, Margaret C. Souders, Jennifer A. Pinto-Martin, Rhonda C. Boyd, Janet A. Deatrick

**Affiliations:** 1 Stefanie Zavodny Jackson, PhD RN, University of Pennsylvania, Philadelphia, PA, USA; The Children’s Hospital of Philadelphia, Philadelphia, PA, USA; 2 Margaret C. Souders, PhD CRNP, University of Pennsylvania, Philadelphia, PA, USA; The Children’s Hospital of Philadelphia, Philadelphia, PA, USA; 3 Jennifer A. Pinto-Martin, PhD MPH, University of Pennsylvania, Philadelphia, PA, USA; University of Pennsylvania Perelman School of Medicine, Philadelphia, PA, USA; 4 Rhonda C. Boyd, PhD, The Children’s Hospital of Philadelphia, Philadelphia, PA, USA; University of Pennsylvania Perelman School of Medicine, Philadelphia, PA, USA; 5 Janet A. Deatrick, PhD RN FAAN, University of Pennsylvania, Philadelphia, PA, USA; The Children’s Hospital of Philadelphia, Philadelphia, PA, USA

**Keywords:** autism spectrum disorder, mothers, depression, self-efficacy, family functioning

## Abstract

**Background:**

Mothers of children with autism spectrum disorder (ASD) report significantly more depressive symptoms, lower maternal self-efficacy, and worse family functioning than mothers of neurotypical children and children with Down Syndrome. There is a need to describe what contributes to high self-efficacy and high family functioning among mothers of children with ASD.

**Aim:**

To understand how mothers of children with ASD describe their emotions, maternal self-efficacy, and family functioning, comparing mothers who screen positive and negative for symptoms of depression.

**Methods:**

In this second phase of a larger sequential explanatory mixed methods study, this qualitative descriptive study was conducted using semi-structured individual interviews with mothers of children with ASD. The data were analyzed by hybrid directed content analysis for a presentation of themes. Those themes were then compared across three groups: mothers who screened positive for depression, mothers who screened negative, and mothers whose screening results changed over time.

**Results:**

Mothers in the group who screened positive for depression described more child behaviors perceived as problematic, higher caretaking demands, maternal self-efficacy dependent on child’s progress and comparisons to others, more passive coping, and poor family communication.

**Conclusion:**

Interventions to improve family processes and maternal self-efficacy may be an important complement to individual therapy for treating maternal depression. Intervening at the family level as soon as the child is diagnosed may also help mitigate maternal depressive symptoms. More research is necessary to understand and develop interventions that can improve the experiences of mothers of children with ASD.

## Introduction

Mothers of children with autism spectrum disorder (ASD) report more stress, anxiety, and depression than fathers of children with ASD, parents of typically developing children, and parents of children with other disabilities (Bispo-Torres et al., 2023; Jackson et al., 2024). Their poor self-reported outcomes are related to the prospect of their children’s lifelong challenging and disruptive behaviors and low social reciprocity, as well as uncertainty about their children’s long-term well-being (Dale et al., 2006; Ebadi et al., 2021; Greenberg et al., 2004; Trew, 2024). These associations have a unique impact on the mothers’ mental health outcomes due to a mother’s dominant role as the primary caregiver and the social stigma of blame for children’s “misbehaviors” (Demirpençe Seçinti et al., 2024; Trew, 2024). Mothers experience exhaustion from the responsibility of managing the child’s ASD on top of the family’s day-to-day life, especially when the mother feels solely responsible for the child’s care (McAuliffe et al., 2019; Safe et al., 2012; Vilanova et al., 2022). Many mothers feel uncertain about how to best care for their child or where to turn for help, leading to feelings of helplessness and frustration (Papadopoulos, 2021). Mothers fear stigma and judgment from others and do not feel they can relate to other parents, leading to isolation from other families and members of their extended family (Bohadana et al., 2021; Papadopoulos, 2021). When Dr. Leo Kanner first wrote about autism in the 1940s, he described “refrigerator mothers” who impede the child’s social and emotional development by being emotionally distant from their children (Silverman, 2012). The effects of this term can still be observed today; mothers who believe they have personal responsibility for their child’s condition report more depressive symptoms (Dale et al., 2006).

Recent research has shown how stress and depression in a mother of a child with ASD can affect relationships among family members and the family’s whole way of life. Mothers of children with ASD experience being less patient with their children, spending less time with their neurotypical children, and worse family coping in general as a result of their high parenting stress (Bohadana et al., 2021; Papadopoulos, 2021). Consequently, neurotypical siblings feel neglected and resentful toward their parents (Papadopoulos, 2021). Parenting a child with ASD can also change the mother’s relationship with her partner, often characterized by neglecting each other’s needs, breakdown in communication, feeling disconnected from each other, and sometimes resulting in separation (Ebadi et al., 2021; Papadopoulos, 2021; Vilanova et al., 2022).

Because of the implications for the health of the child and family as a whole, there is a need to understand mothers’ experiences of depressive symptoms and parenting through the lens of maternal self-efficacy and general family functioning. In a recent quantitative prevalence study among three groups of women—mothers of children with ASD, mothers of children with Down Syndrome, and mothers of neurotypical children—the authors found that mothers of children with ASD report statistically significantly higher rates of positive depression screening, lower maternal self-efficacy, and worse family functioning than the two comparison groups (Jackson et al., 2024). These results indicate that maternal self-efficacy and family functioning may be impactful targets of future interventions for preventing and treating depression in mothers of children with ASD (Jackson et al., 2024).

However, before interventions can be meaningfully designed and implemented, there is a need to describe what mothers of children with ASD, both with and without depressive symptoms, perceive contributes to high self-efficacy and family functioning. This qualitative study sought to explore the mothers’ experiences underlying their reports of maternal self-efficacy and family functioning, comparing how those descriptions may differ based on the presence or absence of clinically significant depressive symptoms. This approach could enable a better understanding among clinicians of how to best anticipate and address this population’s needs.

### Theoretical Framework

This qualitative study was grounded in Bandura’s social cognitive theory and theory of self-efficacy and Teti & Gelfand’s definition of maternal self-efficacy (Bandura, 1986; Teti & Gelfand, 1991). Bandura’s social cognitive theory posits that a person (personal factors) influences and is influenced by their behavior and the environment (Bandura, 1986). In this theory, self-efficacy is an important personal factor that impacts a person’s behavior and is defined as an individual’s feeling of confidence in their ability to perform a specific behavior to achieve a desired outcome. As such, self-efficacy cannot be assessed generally and must be assessed according to a specific behavior. This study measured maternal self-efficacy, which is defined by Teti and Gelfand (1991) as self-efficacy about the mother’s behaviors related to specific childcare activities. Self-efficacy is determined by four factors: prior experience, vicarious experience, coaching and feedback, and physiological and psychological state (Bandura, 1986).

**Figure 1.**
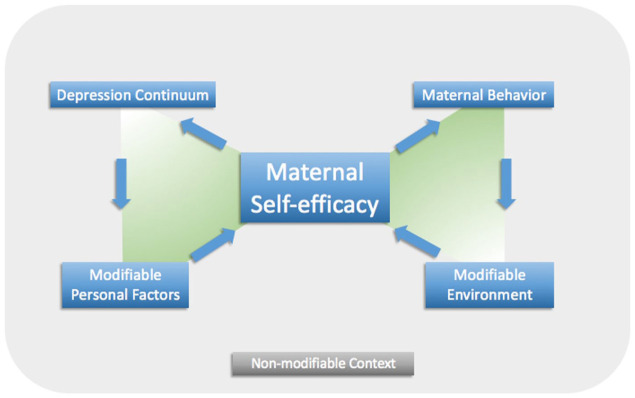
The Maternal Self-Efficacy Model. *Note.* The Maternal Self-Efficacy Model illustrates the relationships among depressive symptoms, self-efficacy, family functioning, and child behavior and served as a framework in designing this study.

To better understand the relationships among maternal depressive symptoms (personal factor), maternal self-efficacy (personal factor), and family functioning (environment), the study team combined Bandura’s social cognitive theory and theory of self-efficacy, under the lens of Teti & Gelfand’s definition of maternal self-efficacy, to create the Maternal Self-Efficacy Model (Figure 1) (Jackson et al., 2024). The Maternal Self-Efficacy Model guided the development of the study’s interview guide and the analytic approach.

### Aim

The purpose of this qualitative study was to understand how mothers of children with ASD describe their emotional states, maternal self-efficacy, and family functioning, comparing mothers who screen positive for depression to mothers who screen negative.

## Method

### Design

This qualitative descriptive research study followed a first, quantitative prevalence study, in a sequential mixed-methods explanatory design study (Creswell & Plano Clark, 2011). The sequential mixed-methods study was approved by the Institutional Review Boards at the Children’s Hospital of Philadelphia and the University of Pennsylvania.

### Participants

The sample was derived from the larger convenience sample of 101 mothers of children with ASD who participated in the first prevalence study (Jackson et al., 2024). Mothers were eligible if they were 18 years old or older, English-speaking, able to provide informed consent, had a biological child younger than 18 years old diagnosed with ASD, and consented to be re-contacted at a later date for this qualitative study. Mothers were excluded if they did not read or speak English, did not live in the same household as the child, if the child had a severe acute condition (e.g., traumatic injury) or complex chronic illness (e.g., cancer or cancer-survivor), or if they did not consent to be re-contacted for the qualitative study. Mothers who completed the interview received a $25 gift card (as a token of appreciation) and a binder with resources for ASD support.

### Sampling Procedures

The study team intentionally sought to sample two groups of women based on the presence or absence of depressive symptoms as determined by their score on the Patient Health Questionnaire-9 (PHQ-9; Spitzer et al., 1999). The first sampling group was mothers who screened positive (PHQ-9 score > 10) for depressive symptoms at the time of the prevalence study (samp-P), and the second sampling group (samp-N) was mothers who screened negative (PHQ-9 score < 10). The study team utilized purposive maximum variation sampling methods to identify potential participants for the qualitative study, seeking heterogeneity in the key variables from the first prevalence study (Jackson et al., 2024). A table was created displaying all eligible participants’ scores on the PHQ-9, Maternal Efficacy Scale (Teti & Gelfand, 1991), and Family Assessment Device (Epstein et al., 1983), along with demographics for the mother, child, and family. Using the table to visualize variation in quantitative scores, risk factors for depression, and minority groups, the study team invited eligible samp-P mothers and samp-N mothers to join the qualitative study. The opportunity to participate in the qualitative study was introduced via e-mail with follow-up phone calls as needed. On average, interviews took place 4 months (range: 2.25–5.25 months) after the participant’s engagement in the prevalence study.

### Setting

The study’s lead investigator coordinated mutually agreeable times and settings for the interviews. Participants were interviewed in their homes or private areas of the hospital, research center, or clinic. The investigator asked that the child not be present in the room for the mother’s interview. If the child was home during a home-based interview, a caregiver (provided either by the parents or the investigator) was present to supervise the child. Participants were informed that the interview might take up to 90 minutes of their time.

### Data Collection

The lead investigator conducted the interviews following the interview guide. The interview guide contained open-ended questions with probes to explore the concepts in the Maternal Self-Efficacy Model (Figure 1): maternal self-efficacy, depression, coping behavior, child behavior and ASD features, and family functioning (Hsieh & Shannon, 2005). The interview guide was adapted according to information collected during each interview (D. G. Willis, Sullivan-Bolyai, et al., 2016).

Following the interview, mothers were asked to complete a PHQ-9 screening questionnaire for a second time. The PHQ-9 asks participants to indicate how often they have experienced symptoms of depression in the past 2 weeks (Spitzer et al., 1999). Each item is scored on a 4-point Likert-type scale, ranging from 0 (*Not at all*) to 3 (*Nearly every day*). A score of >10 was considered a positive screening result and has been validated in adult primary care (sensitivity = 0.74, specificity = 0.91) (Arroll et al., 2010).

### Procedures

The structured 1:1 interviews were conducted from August 2018 to October 2018. The first author recorded memos and field notes during and immediately after the interviews. The interviews were voice-recorded, then transcribed by a professional transcription service. Interviews ended when repeated themes were observed with no new information obtained, indicating saturation of data (Morse, 2015).

All mothers who participated were provided with information about local and national mental health resources. The study followed the protocol published by Lucas and colleagues (2015) for any participants who reported suicidal ideation or behavior during the interview, which included administration of the Columbia-Suicide Severity Rating Scale (C-SSRS) and instructions for care escalation according to C-SSRS results (Lucas et al., 2015).

### Data Analysis

Qualitative data were managed using ATLAS.ti (ATLAS.ti Scientific Software Development GmbH, 2018, version 8.2.4). Interviews were professionally transcribed and then cleaned by a member of the study team. After verification for accuracy, the transcripts were uploaded into ATLAS.ti. Field notes and reflections were incorporated into the transcripts. An audit trail of memos was recorded to document analytical decisions and increase transparency in the analytical process (D. G. Willis, Sullivan-Bolyai, et al., 2016). All data were stored on a secure server.

More than 500 single-spaced pages of transcribed data, reflecting over 1,600 minutes of interviews, were analyzed by the first author with oversight and consultation from a mixed methods expert (J.A.D.) using a hybrid approach to directed content analysis (Proudfoot, 2022). This approach combines deductive and inductive content analysis by both using a priori themes from a theoretical framework and remaining open to any themes present in the data that do not fit the theoretical framework (Proudfoot, 2022). The study team’s coding guide was developed using the concepts of the Maternal Self-Efficacy Model (Figure 1), while remaining open to other themes and subthemes that might emerge from the data.

The content analysis followed the phases outlined by Hsieh and Shannon (2005). The first author performed a close reading of the transcripts to identify and code as many potential themes as possible from the established coding guide. The data were then compared across participants to develop subthemes reflecting variations in the mothers’ experiences and descriptions of the larger themes (Ayres et al., 2003). The within- and across-case analyses were approached using an iterative process. Memos were written to maintain an audit trail of analytic decisions made. A detailed analysis was conducted for each theme to identify the content of the theme as well as how it fit into the overall dataset and in relation to the mother’s description of her experiences.

The quantitative and qualitative data were integrated in the interpretation phase (Creswell & Plano Clark, 2011; O’Cathain et al., 2010). Data were compared using an informational matrix anchored in quantitative depression screening results from the day of the interview, which displayed the quantitative results and qualitative themes together, allowing the authors to recognize themes across the data (O’Cathain et al., 2010; Wendler, 2001). The informational matrix was used first for within-case analysis, where the qualitative themes were summarized separately for each case. Then the themes were compared across cases to identify differences in qualitative results based on the depression score. An informational matrix enhances methodological rigor and transferability of results.

The study adhered to guidelines described by Guba (1981) to address the trustworthiness of qualitative research. The research team consisted of a registered nurse, a nurse researcher, a nurse practitioner in child psychiatry, a child psychiatrist, and an epidemiologist. While members of the research team treated patients at the health system where the study was performed, the study participants were not patients of any staff member, and members with an active clinical practice only interacted with de-identified data. The first author conducted all interviews. Credibility of the data was assured by debriefing after the interviews. The interviewer discussed the interview content with academic advisors with research and clinical expertise in this area of nursing practice (M.C.S.) and with expertise in mixed methods (J.A.D.). Maximum variation sampling was performed, and thick descriptive data were collected to address the transferability of results. An audit trail was maintained during the data analysis process to demonstrate dependability and confirmability. In addition, the investigator took a reflexive approach in the audit trail to contribute to the confirmability of the results (Guba, 1981).

## Results

The study was introduced to 39 mothers: 21 in the samp-P group and 18 in the samp-N group. Following the previously described sampling and study procedures, we enrolled 21 participants: 14 mothers in the samp-P group, and seven mothers in samp-N group.

Following the interviews, the participants all completed a second PHQ-9 questionnaire. We found that some of the mothers’ screening results changed from the time of the prevalence study. Specifically, eight mothers in samp-P screened negative on interview day, and nine mothers in samp-*N* screened positive on interview day. These findings were incorporated into the study’s design and led us to create three groups for the analysis (from the initial design of two groups). The three groups include Group P (positive): mothers who screened positive at both encounters (*n* = 6); Group N (negative): mothers who screened negative at both encounters (*n* = 6); and Group C (changed): mothers whose screening result changed between the two time points (*n* = 9).

Demographic information for the total sample and the three groups is presented in Table 1. The qualitative themes for the sample and each of the three groups are described below. The themes are then illustrated by a case exemplar from each of the three groups.

**Table 1.** Sample Demographic Characteristics.

	Total (*N* = 21)	Group P (*n* = 6)	Group N (*n* = 6)	Group C (*n* = 9)
Mother characteristics	*n* (%)			
Age (*M*)	38.1	35.3	42	37.3
Race				
White	13 (61.9%)	4 (66.7%)	4 (66.7%)	5 (55.6%)
Multiracial	1 (4.8%)	1 (16.7%)	0	0
Hispanic	3 (14.3%)	1 (16.7%)	0	2 (22.2%)
Black/African American	1 (4.8%)	0	0	1 (11.1%)
Asian	3 (14.3%)	0	2 (33.3%)	1 (11.1%)
Marital status				
Now married	12 (57.1%)	3 (50%)	4 (66.7%)	5 (55.6%)
Never married	5 (23.8%)	2 (33.3%)	1 (16.7%)	2 (22.2%)
Divorced/Separated	4 (19.0%)	1 (16.7%)	1 (16.7%)	2 (22.2%)
Education				
High school diploma	1 (4.8%)	0	0	1 (11.1%)
2-year/Some college	4 (19.0%)	2 (33.3%)	1 (16.7%)	1 (11.1%)
Bachelor’s degree	10 (47.6%)	3 (50%)	2 (33.3%)	5 (55.6%)
Graduate degree	6 (28.6%)	1 (16.7%)	3 (50%)	2 (22.2%)
Employment				
Employed full-time	10 (47.6%)	4 (66.7%)	4 (66.7%)	2 (22.2%)
Employed part-time	8 (38.1%)	2 (33.3%)	1 (16.7%)	5 (55.6%)
Homemaker	2 (9.5%)	0	1 (16.7%)	1 (11.1%)
Out of work or unable to work	1 (4.8%)	0	0	1 11.1 (%)
Annual household income				
Less than $24,999	1 (4.8%)	0	0	1 (11.1%)
$25–49,999	5 (23.8%)	2 (33.3%)	2 (33.3%)	1 11.1 (%)
$50–74,999	7 (33.3%)	2 (33.3%)	0	5 (55.6%)
$75–99,999	1 (4.8%)	0	0	1 (11.1%)
$100,000 or more	7 (33.3%)	2 (33.3%)	4 (66.7%)	1 (11.1%)
Personal mental health history				
No diagnosis	9 (42.9%)	2 (33.3%)	3 (50%)	4 (44.4%)
Depression diagnosis	11 (52.4%)	4 (66.7%)	2 (33.3%)	5 (55.6%)
Anxiety	1 (4.8%)	0	1 (16.7%)	0
Child characteristics				
Child age in years	7.8	6.8	7.8	8.3
Child’s gender				
Male	17 (81.0%)	4 (66.7%)	5 (83.3%)	8 (88.9%)
Female	4 (19.0%)	2 (33.3%)	1 (16.7%)	1 (11.1%)
Family characteristics				
Family history of depression	8 (38.1%)	3 (50%)	3 (50%)	2 (22.2%)
Partner lives in the home	14 (66.7%)	4 (66.7%)	5 (83.3%)	5 (55.6%)
Partner mental health history				
No diagnosis	17 (81.0%)	3 (50%)	6 (100%)	8 (88.9%)
Depression diagnosis	3 (14.3%)	2 (33.3%)	0	1 (11.1%)
Other	1 (4.8%)	1 (16.7%)	0	0
Multiple children with neurodevelopmental dx	4 (19.0%)	2 (33.3%)	1 (16.7%)	1 (11.1%)

*Note.* dx = diagnosis.

### Qualitative Themes

The qualitative data contained five major themes: the mothers’ emotional states, the child’s behavior and autistic traits, self-efficacy, coping behaviors, and family functioning. All themes were consistent with our coding guide based on the Maternal Self-efficacy Model. As part of our hybrid directed content analysis approach, we remained open to themes outside of our a priori coding guide, but no other themes emerged.

The narrative accounts of mothers’ emotions and mood contained several subthemes, including changes in mood, depressive symptoms, blame and responsibility, social isolation, worries and anxiety, and personal sacrifices. In describing their child with ASD, they discussed diverse behaviors and characteristics and how they change over time, the care their child’s ASD requires, their hopes and expectations for their child’s progress, and their relationship with their child. Mothers mentioned several sources of their self-efficacy, including how they learned to handle certain demands. In discussing what they do to make themselves feel better and take care of themselves, mothers described a variety of behaviors and coping strategies, which are categorized into active and passive coping strategies. Mothers described the roles and relationships among different family members, including nuclear and extended family, and how they communicate and support each other. They also described how having a child with ASD impacts the family as a whole, including adapting to create their own version of a normal family life and the sacrifices the family makes to adjust.

#### Group P, Positive Depression Screening

The narratives of the mothers in Group P illustrated low mood states that were persistent, regardless of external factors. Their low mood, loss of interest, fatigue, and sense of social isolation could impair functioning and make it difficult to be optimistic about the future. They also discussed feeling a sense of lost identity, which is connected to low self-worth and either not enjoying or not having time for self-care activities. The Group P mothers want their children to reach certain goals, but are not confident they will achieve them due to severe problem behaviors and high care-taking demands. Mothers express guilt and self-blame when their child acts out or does not progress as expected, often internalizing this as a reflection of their own inadequacy. Low self-efficacy also results from comparing themselves to other mothers and comparing their child to other children, both those with typical development and those with ASD. The Group P mothers described a sense of inferiority when they see others’ success. They cope actively by making environmental changes (e.g., safety alarms, removing furniture), making efforts to be mindful and present, and suppressing competing activities (consciously focusing on one issue at a time in the presence of multiple competing demands). However, they described feeling guilty if they were unable to do other tasks as a result of focusing on the problem at hand. They also described passive coping strategies: avoidance of stressful situations, suppression of emotions to get through the day, and mental disengagement (e.g., denial, distractions, giving up).

The Group P mothers’ narratives revealed that their families have not adapted well to their child’s ASD diagnosis and symptoms. These mothers expressed a desire for normalcy and focused on what makes their family abnormal. The parenting roles are imbalanced, and the burden of responsibility lies with the mother. Communication among family members is strained, and mothers do not seek emotional support from their partners. This intensifies their feeling of isolation; they feel isolated not only from the outside world and their peers, but also from other members of the family.

#### Group N, Negative Depression Screening

The mothers in Group N described generally good moods with some feelings of stress and being overwhelmed, which can fluctuate depending on the day. These mothers’ sense of responsibility for their child’s ASD results not in guilt, but pride when their child succeeds and progresses. They recognize that their family may be different, but are still able to feel connected with other parents. They reject judgmental comments from others that insinuate they are to blame for any part of their child’s ASD. Some child behaviors, such as stimming, repetitive speech, and restricted interests, are accepted as part of ASD, perceived as problematic mainly if they interfere with the child’s functioning, learning, and social interaction.

Like the Group P mothers, the Group N mothers often worry about their child’s safety and future, but what links these mothers is a sense of acceptance—either of the uncertainty about their child’s future, their low expectations regarding that future, or even their high expectations. Group N mothers base their self-efficacy on their education, maternal instinct, and feedback from others. Despite high self-efficacy, the mothers in this group express uncertainty about whether they are doing the best thing for their child. They described active coping strategies, including self-care, acceptance, advocacy activities, and recognizing and celebrating progress. The families have adapted to their child with ASD; they have created their own “normal” and enjoy activities together as a family. The group N mothers and their partners take a team approach with defined and balanced roles, shared responsibilities, and open communication.

#### Group C, Depression Screening Result Changed

The mothers in Group C described a wide range of emotions, from happy to severely depressed; these feelings can fluctuate depending on external stressors such as the child’s behavior, work, judgment from others, and societal expectations. Themes regarding child behavior and autistic traits, and maternal self-efficacy were mixed. The severity of their child’s behaviors and traits ranged from moderate to profound, with varying degrees of acceptance regarding expectations for the future. Mothers’ self-efficacy in childcare activities varied; sources of self-efficacy included recognizing a child’s progress, comparing themselves to others, feedback from others, and maternal instinct. The narratives revealed coping strategies including acceptance, religion, humor, and seeking emotional support from outside the nuclear family, including neighbors, other mothers, and therapists. Family functioning and adaptation were mixed in this group as well. The mothers hold most of the childcare responsibility, either because they do not have a partner to share the responsibilities or because they feel they know what is best. The practical support provided by extended family, neighbors, and health providers plays a large role for these mothers.

### Exemplar Cases

#### Case Exemplar, Group P Mother

Mom A is 34 years old and married. She and her husband have a 5-year-old son with ASD. Mom A works full-time, and her husband works two jobs. Both her parents and her in-laws live nearby and provide support. She has a history of depression and sought therapy in college, but has not had treatment since then. Her Phase 1 and 2 PHQ-9 scores were 15 and 12, respectively, and she was tearful throughout the interview. She describes her son as “a happy, smiley, funny little boy . . . He is just the best kid.” His autism is profound; he is minimally verbal, stims (performs self-stimulating behavior) constantly, is selective about food, and elopes from the home. Mom A is concerned about his safety, and the family has alarms throughout their house and an autism alert sticker on their car. The child’s school program is based on applied behavior analysis (ABA), and they are focusing now on using ABA techniques for toilet training. She struggles with setting realistic goals because “I don’t want to get my hopes up and have them dashed, but I also don’t want to be negative or . . . hinder his progress . . .” When asked how she feels throughout a normal week, she described, “. . . a never-ending, crushing feeling of not doing enough . . . Pretty much at home, I’m miserable.” She feels she cannot do the activities with him that she enjoyed as a child, and she feels that she is not meeting the expectations she had for herself as a parent. She has trouble accepting his differences, and “[wants] him to be able to speak and tell [her] that he’s sick, or hear him say, ‘I love you, mommy.’” She feels guilty and wonders what she did that could have caused his ASD. She compares herself to “other moms who know the acronyms, and . . . how to get what their child needs . . . I don’t know how to do any of that . . . I always leave feeling like the dumb one in the group . . .” She tends to keep her feelings to herself; talking to her husband is “not a real dialogue about all my fears.” She will occasionally reach out to friends and other parents of children with ASD for emotional support, information, and fun nights out. She tries to “cheer for any and all gains” her child exhibits and focuses on the current issues instead of getting overwhelmed about the future. Going to work is her respite when she does not have to think about all her worries. She does not ask her husband to help or share childcare responsibilities because “it’s frustrating to have to . . . double-check everything to make sure, again, even when you delegate, that it gets done properly.” Her husband has not fully accepted their son’s diagnosis and has not made efforts to learn about ASD and his specific needs, which causes strain in their relationship. They are in marital counseling and have decided not to have more children due to their son’s needs.

#### Case Exemplar, Group N Mother

Mom B is 35 years old, married, 7 months pregnant with twins, and has two sons, ages 4 and 6. She is a stay-at-home parent, and her husband works full-time. She has no personal history of depression, but she does have a family history (mother). Her Phase 1 and 2 PHQ-9 scores were 1 and 6, respectively. Her husband’s family lives nearby. She describes her older son, who is diagnosed with ASD, as “amazing” and “a snuggler.” He is fully verbal, has sensory sensitivities, specific interests, a need for routines, and literal understanding. He has expressed interest in engineering like his parents, and Mom B is confident that he will be able to achieve this goal. She describes herself as an outgoing, social, and easy-going person who thrives on stress. She was bullied when she was younger and is worried about her son being bullied. She has felt judged for a variety of decisions she has made as a mother, but has realized, “It’s just different, the different things that work . . . I very much learned I do not care what other people’s opinions are.” She is confident that she knows what her son needs based on previous trial and error. Her self-efficacy in handling temper tantrums and social problems was built during Relationship Development Intervention therapy; the therapist would model strategies and provide feedback, “like ‘But you did this really well . . . Try it again.’ Two or three times of doing that, and I would start to feel more comfortable.” The hardest part for her is “juggling different kids’ needs, not trying to focus on one or the other . . .” When she decided to leave her job after her second son was born, she joined a local mom’s group, which she describes as “lifesavers” and a “no-judgment zone.” She can reach out to that network for instrumental support and information. She is an “eternal optimist,” recognizes and celebrates signs of progress in her child, and accepts that “nobody’s perfect, and I’m not a fan of being normal or regular either . . .” Her family has adapted well to the ASD diagnosis, and they value “family dinners, spend[ing] time together, play[ing].” She and her husband share responsibility for making important decisions. During her pregnancy, her husband has taken on more household responsibilities so she can rest. Her sons have “that special brotherly connection,” and she loves their close bond.

#### Case Exemplar, Group C Mother

Mom C is 32 years old, married, and has a 5-year-old son with ASD. She is a stay-at-home parent, and her husband works full-time. Both her parents and in-laws live nearby, but she has a strained relationship with her parents and considers only her in-laws a source of support. She has a personal history of depression and anxiety and is currently treated with medication. Her Phase 1 and 2 PHQ-9 scores were 13 and 8, respectively. She reports that she has been feeling good lately, since she decided to take her son out of school. Fighting with the school district was a major source of stress, and after a disastrous first day of school this year, she decided to homeschool him. She had already decided to leave her job because of her son’s behaviors, and because she did not trust anyone else to manage his tendency to elope. He has co-occurring oppositional defiant disorder, has phrased speech, can be aggressive, and seeks sensory input. She has set and accepted what she believes to be a realistic goal for him to “be a good human.” She has trouble sleeping, occasional anxiety attacks, and worries about his future and where he will live. She used to feel guilty because “Friends and family are saying . . . he’s spoiled, or you’re giving him too much tablet time. He’s watching TV too much.” Her instincts have since been validated when a neurologist acknowledged that the technology was a way to reach him. She is still figuring out how to manage his outbursts, but they have been able to enjoy their time together traveling over the summer and now at home. She is grateful that she is in “such a good spot now, and my life is crazy. You have to accept it . . . life is nowhere I expected it to be, but it’s exactly where I would want it to be.” Her coping includes humor and seeking emotional support from her husband and a variety of neighbors and friends, stating, “We just joke about it, and we drink, and we joke about it, and we see our friends, and then we have fun.” Her family is working on adjusting to the child’s behaviors. Her husband has obsessive-compulsive disorder and “explosive anger disorder,” and he is often triggered by the inevitable messes in the house. Mom C feels bad asking her husband for help because he works all day. Their goal as a family is to be happy, and while recognizing their imperfection, she feels she and her husband were “. . . meant to be his parents . . . No we’re not perfect. Dear God, no, but we are able to do it.”

## Discussion

The results of this qualitative study provide new insight into the diverse experiences of mothers of children with ASD and their emotional state, self-efficacy, and general family functioning. The study’s diverse sample, including various characteristics that are known to be associated with depression, enabled us to capture a wide range of experiences of mothers of children with ASD. This is crucial, as it is the first study to qualitatively explore how mothers’ descriptions of maternal self-efficacy and family functioning differ based on the presence of chronic and situational depressive symptoms. The themes are not unique to this population and may be true for any mother or family, but the comparison of themes based on presence, absence, and change of depressive symptoms illuminates the experiences of motherhood and impacts of symptoms of depression among mothers of children with ASD.

Our family functioning and coping subthemes are consistent with previous studies of families of children with ASD. Coping strategies described by our sample echo previous qualitative descriptions of coping in this population, including the importance of knowledge, self-care, seeking outside supports, and recognizing moments of joy (Kuhaneck et al., 2010). Compared with families without a child with ASD, families of children with ASD report more chaotic adaptability levels, which reflects a lack of consistency and constantly changing rules and roles (Altiere & von Kluge, 2009). The family’s level of emotional bonding influences the family’s coping, specifically whether the mother seeks support from friends or family members, which in turn is correlated with maternal stress (Altiere & von Kluge, 2009; Pardo-Salamanca et al., 2024). Furthermore, depressive symptoms have been associated with maladaptive coping strategies such as behavioral and mental disengagement, denial, and self-blame, and this association is reflected in the coping subthemes described by Group P (Benson, 2010; Smith et al., 2008; K. D. Willis, Timmons, et al., 2016).

The relationships we observed among maternal self-efficacy, child behavior, and family functioning are also consistent with findings in families of children with other chronic conditions. Among caregivers of adolescent and young adult brain tumor survivors, caregiver competence, which is similar to self-efficacy, is directly predicted by the child’s health and family functioning (Deatrick et al., 2014). Of note, it is the caregiver’s *perception* of the child’s health that predicts competence; professional ratings of health were not significant (Deatrick et al., 2014). A similar finding is reflected in the narratives in this study, with mothers’ perceptions of child progress contributing to a higher sense of self-efficacy. These relationships are important to consider when developing interventions to address maternal depression and self-efficacy using a combined individual and family approach.

The results of this qualitative study highlight the role of time and cross-sectional measures of symptoms. Depressive symptoms, self-efficacy, and child behaviors and traits can all change over time. In our study, about 4 to 5 months passed between the initial prevalence study and the interviews, and because we used a short-term screening tool to measure depression, we saw nine (9/21, 43%) mothers’ depressive screening results change over time, making it necessary to analyze the data in three groups. While this was unanticipated, it allowed us to explore situational or episodic depressive symptoms. Screening participants after the interview, the mothers in Group C were able to identify external reasons for the change in their stress levels and depression symptoms. These included changes in work stress, return to work, initiation or return to therapy, and change in the child’s schooling. While some of these external changes were also reported in Group P, the mothers in Group C crossed the threshold for clinically significant depressive symptoms at the time of the interview. The change in screening result indicates that they were experiencing significant fluctuations in symptoms, while the mothers in Group P experienced more stable or chronic depressive symptoms. The thematic differences found in the analysis support the assertion that the Group C mothers’ depressive symptoms were situational. In Group C, the mothers’ mood and confidence seemed more sensitive to the environment, ranging widely depending on their child’s behavior and progress, daily demands, and work or school stressors.

To truly study the impact of time and causal associations between the environment and maternal depression, longitudinal studies must be performed. Results of a previous longitudinal study with mothers of children with ASD show that maternal distress, including both depression and anxiety, increased over the duration of the study, from the child’s birth to age 14 (May & Williams, 2024). While there was an overall increasing trend, the authors observed a peak in maternal distress at the child’s age 6, which was the average age of ASD diagnosis. This supports our finding that some mothers’ depressive symptoms may be significantly impacted by situational factors such as the increased demands placed on a mother around the time of a child’s ASD diagnosis. Furthermore, changes in mothers’ depressive symptoms over time were predicted by parenting efficacy, maternal anxiety, and child behavior (Carter et al., 2009; Roubinov et al., 2023). Given the important role of family processes in this study, future longitudinal studies should include measures of family functioning to determine its role in predicting change in depressive symptoms.

Family processes offer a novel target for addressing depression in this population. Mothers in Group P described poor family adaptation and communication, which was often a source of distress. Families of children with ASD have described what it means for their family to be healthy and what factors impact family health. Smith and McQuade (2021) interviewed mothers, fathers, and their children with ASD and found that accepting imperfection, having social supports, and knowing and understanding the needs and likes/dislikes of each family member and the family as a whole contributed to family health. In a study of parents of children with ASD, fathers’ anxiety and depression augmented the effect of the child’s impairments on mothers’ anxiety and depression (Li et al., 2022). This interaction between mothers’ and fathers’ mental health is reflected in the narrative accounts as well as the group differences in partner mental health history. Family therapy to address issues like communication and coping may be an important complement to individual therapy for treating maternal depression and traditional therapies for ASD. However, more research is needed to explore the causal direction of the relationship between family processes and maternal depression. The timing of the intervention should also be considered; addressing family issues as early as the time of ASD diagnosis may help mitigate maternal depression and improve outcomes for all family members.

The findings of this study support a family approach to caring for a child with ASD. Treatment plans should not only focus on the child’s behavior and ASD features; they should consider the roles and well-being of all members of the family and household. Most treatment currently relies on parents to deliver and evaluate therapies by implementing behavior strategies, administering medications, and reporting on behaviors and symptoms. However, prior studies show that parent anxiety and depressive symptoms are related to their implementation of behavior strategies and reporting of their child’s behaviors (Osborne et al., 2008; Zhou et al., 2019). The themes and narratives in this study demonstrate that family dynamics and maternal self-efficacy also affect the performance of a treatment plan and decision-making regarding the child’s care, which has a direct impact on the child’s outcomes. Our findings illustrate a feedback loop where a parent’s perceptions of those outcomes can impact their motivation to continue with the treatment plan, their view of themselves as a parent, their relationships with others, and their overall mental health. Intervening at the family level can break this cycle and pave the way for better outcomes for the child and all family members.

### Limitations

This study faces several limitations. First is the use of the PHQ-9 to measure depressive symptoms. The PHQ-9 is a screening tool and is not sufficient to diagnose depression. In addition, using the PHQ-9, we were only able to measure depressive state, not depressive trait. For this reason, mothers’ depressive screening results from the first prevalence study to this qualitative study were susceptible to change, limiting our ability to compare the qualitative data based on screening results. A measure of depressive trait would capture more long-term trends in depressive symptoms, potentially allowing a deeper qualitative analysis of differences between those who are at high risk and low risk for depression. To avoid bias in the qualitative data, the PHQ-9 was completed after the interview; however, it is possible that the PHQ-9 answers were impacted by the interview being therapeutic or because the mothers completed the survey in the presence of the interviewer. For a few mothers in Group C, their changed depression screening result was not explicitly explained by situational changes. There was some discordance between interview themes and subsequent PHQ-9 in Group C, which warrants further investigation using an interpretive narrative approach. Future studies of this kind should consider using trait measures of depression, such as the Maryland Trait and State Depression Scale (Chiappelli et al., 2014), or even a concurrent design.

Second, this study only included mothers, but asked about family functioning; so, the understanding of family dynamics and child behavior is limited to the mother’s perspective. Quantitative measures of these factors are impacted by maternal depression, so it is fair to conclude that the qualitative data may be impacted as well. Our understanding of these factors can be enhanced by exploring the perspectives of partners, siblings, children with ASD, and other important family members.

Third, while our sample was purposely chosen to be as diverse as possible in the factors that are associated with maternal depression in this population, the majority of our sample was White (61.9%), had at least a bachelor’s degree (76.2%), and were married or partnered (66.7%). Given the potential impact of social determinants on maternal mental health, future studies should continue to include measures of social determinants such as economic stability and access to health care and other necessary resources. In addition, our understanding of the experiences of mothers of children with ASD would benefit greatly by including more diverse samples in future studies.

Last, the coding and interpretation of data were performed by one individual; however, the analysis was done under the guidance and direction of experts with a reflexive approach. Regardless, it needs to be acknowledged as a limitation and a potential source of bias in the analysis.

## Clinical Implications for Nursing Practice

Just as every child with ASD is unique, every family is unique in its structure, dynamics, and coping. Some families adapt well and thrive in the setting of an ASD diagnosis, but for others, an ASD diagnosis may reveal weaknesses in the family’s coping and functioning. Nurses can educate families about the important role the family’s overall functioning can play in the health of each family member, especially the child’s clinical outcomes and the mother’s mental health. Nurses can empower family members to talk about their experiences as a family member of a child with ASD, both the positive and the negative. As the nurse or nurse practitioner builds rapport with the family, the family’s strengths and weaknesses may become evident, but there are also cases where a family’s struggles are not so obvious or may change over time. For this reason, routine depression screening and assessment of family functioning in practices that provide care for children with ASD would be extremely helpful in addressing this problem. As these results can change over time, it is recommended to screen at every encounter and track results throughout the course of care. Nurses can then advocate for the appropriate referrals to individual treatment for parental depression and family therapy in the interest of providing the highest quality care for families of children with ASD.

## Conclusion

Mothers’ emotional states, child’s behavior and autistic features, self-efficacy, coping behaviors, and family functioning revealed subthemes of changes in moods, blame and responsibility, social isolation, worries, personal sacrifices; change in the child’s behaviors and caretaking demands, expectations for the child’s future; sources of self-efficacy; active and passive coping strategies; the roles and relationships among different family members, family adaptation, and family sacrifices. The description of mothers’ experiences differs based on the presence of clinically significant depressive symptoms. Mothers in Group P described (a) more depressive symptoms and impact on functioning; (b) more child behaviors perceived as problematic, higher caretaking demand, and deeper uncertainty regarding expectations for the child’s future; (c) self-efficacy based on the child’s progress and comparing themselves to other mothers; (d) more passive coping; and (e) imbalanced parenting roles and poor family communication. On the other hand, mothers in Group N described (a) generally good mood with more variation in mood states; (b) fewer child behaviors perceived as problematic, lower caretaking demands, and acceptance of the child’s potential; (c) self-efficacy based on feedback from others and maternal instinct; (d) more positive thinking; and (e) balanced parenting roles and better family communication. Nurses can play a crucial role in providing high-quality care for families of children with ASD by providing education to families, empowering parents, performing routine screening, and advocating for the appropriate referrals to treatment.

## JAPNA: 32.2

**Title:** The experience of depression, self-efficacy, and family functioning among mothers of children with autism spectrum disorder: A mixed methods study

**Authors:**

Stefanie Zavodny Jackson, PhD, RN

Margaret C. Souders, PhD, CRNP

Jennifer A. Pinto-Martin, PhD, MPH

Rhonda C. Boyd, PhD

Janet A. Deatrick, PhD, RN, FAAN

**Disclosures:**

The APNA planner and authors have no relevant financial relationships with ineligible companies or off-label uses to disclose.

**Target Audience:** RN, APRN

**Learning Outcome:**

By the end of this article, the reader will be able to:

1) Identify two differences in the themes described by mothers who screen positive for depression verses negative for depression.

**Key Takeaways:**

1) Mothers of children with ASD should be screened for depression at each office visit.
2) Nurses can help support mothers experiencing depression by promoting active coping strategies, increasing maternal self-efficacy through education and feedback, and addressing the functioning of the family unit.

**Cost:**

There is no fee for Nursing Continuing Professional Development (NCPD) contact hours.

**Nursing Continuing Professional Development Information:**

1 contact hour. To receive contact hours, you must read the entire article, complete a reflection activity, an evaluation, and the honor statement. Once you have completed the activity, evaluation, and honor statement, your nursing continuing professional development certificate will be generated online and available for immediate printing. Credit cannot be earned unless all components of the program are completed.

**The ability to earn contact hours for this article expires March 31, 2028.**

Go to https://www.apna.org/JAPNACEMarch2026 to access the reflection activity, evaluation, honor statement, and certificate.

*The American Psychiatric Nurses Association is accredited as a provider of nursing continuing professional development by the American Nurses Credentialing Center’s Commission on Accreditation.*
